# Supplementary material for: Comparative genomics provides new insights into the diversity, physiology, and sexuality of the only industrially exploited tremellomycete: Phaffia rhodozyma
Source: BMC Genomics. 2016 Nov 9;17:901. doi: 10.1186/s12864-016-3244-7 (PMC5103461; doi:10.1186/s12864-016-3244-7)
Supplement: Additional file 6: — List of orphan genes with links to PFAM (related to Additional file 1: Table S1). (ZIP 1428 kb) [file 12864_2016_3244_MOESM6_ESM.zip › PFAM_UNIQUE/G02416_P.html]

#                                                                            --- full sequence --- -------------- this domain -------------   hmm coord   ali coord   env coord
  
# target name        accession   tlen query name           accession   qlen   E-value  score  bias   #  of  c-Evalue  i-Evalue  score  bias  from    to  from    to  from    to  acc description of target
  
#------------------- ---------- ----- -------------------- ---------- ----- --------- ------ ----- --- --- --------- --------- ------ ----- ----- ----- ----- ----- ----- ----- ---- ---------------------
  
G02416\_P             -            365 CRS1\_YhbY            PF01985.16    84     0.061   12.0   0.4   1   1   3.1e-05      0.19   10.5   0.4    30    74   292   336   285   341 0.88 - CRS1 / YhbY (CRM) domain  
G02416\_P             -            365 DUF4026              PF13218.1    323     0.034   11.7   0.2   1   1   9.4e-06     0.056   11.0   0.2    39   150   178   290   165   299 0.74 - Protein of unknown function (DUF4026)  
G02416\_P             -            365 DUF4162              PF13732.1     84       2.4    7.4   6.2   1   3      0.33     2e+03   -1.9   0.1    13    25    61    73    51   103 0.54 - Domain of unknown function (DUF4162)  
G02416\_P             -            365 DUF4162              PF13732.1     84       2.4    7.4   6.2   2   3   0.00026       1.5    8.0   0.1    31    67   182   218   169   223 0.80 - Domain of unknown function (DUF4162)  
G02416\_P             -            365 DUF4162              PF13732.1     84       2.4    7.4   6.2   3   3     0.031   1.8e+02    1.4   0.4    29    70   291   338   276   354 0.66 - Domain of unknown function (DUF4162)  
G02416\_P             -            365 DUF677               PF05055.7    336     0.067   10.5   1.7   1   2    0.0024       7.2    3.8   0.1   141   178   273   311   232   317 0.84 - Protein of unknown function (DUF677)  
G02416\_P             -            365 DUF677               PF05055.7    336     0.067   10.5   1.7   2   2    0.0007       2.1    5.6   0.2     8    41   326   359   320   362 0.90 - Protein of unknown function (DUF677)  
G02416\_P             -            365 GldM\_N               PF12081.3    194      0.35    9.0   2.7   1   1   0.00047      0.94    7.6   2.7     9    90   272   352   267   361 0.83 - GldM N-terminal domain  
G02416\_P             -            365 Nbl1\_Borealin\_N      PF10444.4     59     0.028   12.5   2.9   1   2      0.17   4.9e+02   -1.1   0.1    15    28    59    72    52    78 0.78 - Nbl1 / Borealin N terminal  
G02416\_P             -            365 Nbl1\_Borealin\_N      PF10444.4     59     0.028   12.5   2.9   2   2   1.2e-05     0.034   12.2   0.7     8    38   313   343   313   345 0.94 - Nbl1 / Borealin N terminal  
G02416\_P             -            365 Nup88                PF10168.4    717       1.1    5.7   4.4   1   1   0.00082       1.6    5.1   4.4   591   661   269   340   237   360 0.81 - Nuclear pore component  
G02416\_P             -            365 Sec8\_exocyst         PF04048.9    142       9.3    4.6   7.4   1   3      0.26   2.2e+02    0.1   0.1    82   101    56    75    49    84 0.79 - Sec8 exocyst complex component specific domain  
G02416\_P             -            365 Sec8\_exocyst         PF04048.9    142       9.3    4.6   7.4   2   3      0.33   2.8e+02   -0.3   0.1    31    79   212   260   203   291 0.73 - Sec8 exocyst complex component specific domain  
G02416\_P             -            365 Sec8\_exocyst         PF04048.9    142       9.3    4.6   7.4   3   3    0.0039       3.3    6.0   1.2    39    96   302   359   288   361 0.83 - Sec8 exocyst complex component specific domain  
G02416\_P             -            365 TroA                 PF01297.12   256       2.3    6.1   5.2   1   1   0.00091       5.5    4.8   5.2    90   167   188   294    40   362 0.76 - Periplasmic solute binding protein family
